# Seeking help in times of economic hardship: access, experiences of services and unmet need

**DOI:** 10.1186/s12888-017-1235-0

**Published:** 2017-03-03

**Authors:** M. C. Barnes, J. L. Donovan, C. Wilson, J. Chatwin, R. Davies, J. Potokar, N. Kapur, K. Hawton, R. O’Connor, D. Gunnell

**Affiliations:** 1 School of Social and Community Medicine, University of Bristol, Bristol, BS8 2PS UK; 2 University of Salford, Salford, UK; 3 Centre for Suicide Prevention, University of Manchester, Manchester, UK; 4 Centre for Suicide Research, University of Oxford, Oxford, UK; 5 Suicidal Behaviour Research Laboratory, University of Glasgow, Glasgow, UK

**Keywords:** Help-seeking, Recession, Services, Need, Experiences, Qualitative, Mental health, Self-harm

## Abstract

**Background:**

Economic recessions are often accompanied by increased levels of psychological distress and suicidal behaviour in affected populations. Little is known about the experiences of people seeking help for employment, financial and benefit-related difficulties during recessions. We investigated the experiences of people struggling financially in the aftermath of the Great Recession (2008-9) - including some who had self-harmed - and of the frontline support staff providing assistance.

**Methods:**

Interviews were conducted with three groups of people in two cities: i) people who had self-harmed due to employment, financial or benefit concerns (*n* = 19) (‘self-harm’); ii) people who were struggling financially drawn from the community (*n* = 22), including one focus group) (‘community’); iii) and frontline staff from voluntary and statutory sector organisations (e.g., Job Centres, Debt Advice and counselling agencies) providing support services to the groups (*n* = 25, including 2 focus groups) (‘service providers’). Data were analysed using the constant comparison method.

**Results:**

Service provision was described by people as confusing and difficult to access. The community sample reported considerably more knowledge and access to debt advice than the participants who had self-harmed – although both groups sought similar types of help. The self-harm group exhibited greater expectation that they should be self-reliant and also reported lower levels of informal networks and support from friends and relatives. They had also experienced more difficult circumstances such as benefit sanctions, and most had pre-existing mental health problems. Both self-harm and community groups indicated that practical help for debt and benefit issues would be the most useful – a view supported by service providers - and would have particularly helped those who self-harmed.

**Conclusion:**

Interventions to identify those in need and aid them to access practical, reliable and free advice from support agencies could help mitigate the impact on mental health of benefit, debt and employment difficulties for vulnerable sections of society.

## Background

Economic recessions are usually accompanied by increases in unemployment and economic hardship and also political measures, such as austerity, to hasten economic recovery [1]. These are associated with rises in suicide and suicide attempts [2–5]. During the recent recession (2008-9) suicides rates increased across most European and American countries, particularly in those where there were higher levels of job loss [3]. English regions with the largest rises in unemployment had the largest increases in suicide, particularly amongst men [2, 6] and the previously downward trend in young male suicide rates (16-34 years) halted around 2006 [4]. In Ireland 5 years of economic recession and austerity had a significant negative impact on suicide in men and self-harm in both sexes [5]. Recent research indicates that some austerity measures have adversely affected suicide rates [7] and that Government spending on active labour market programmes can mitigate the impact of unemployment on the risk of suicide [8].

People with pre-existing mental health problems are the most vulnerable to debt and other financial difficulties [9, 10]. Whilst causality is hard to establish [9], economic hardships resulting from austerity measures can act as a trigger to suicidal behavior amongst vulnerable populations [10]. However, even in times of prosperity, job loss and debt are associated with depression and suicide risk [11, 12]. The majority of this work has been epidemiological in nature. The very small body of qualitative literature describing the impact of austerity measures (difficult economic conditions created by government measures to reduce public expenditure), highlights their impact on mental health as people try to manage on increasingly limited budgets and benefits [13, 14].

Few qualitative studies have investigated the experience of people affected by job loss, financial difficulties, housing and benefit worries as they seek help for their practical and mental health needs; such studies are needed to inform the development of appropriate interventions. The research to date has often reported on the experiences of particular populations’ with, and barriers to, accessing services for debt, welfare or employment advice (e.g., [10, 15, 16]). Most studies report patchy activity across organisations, with a call for co-ordinated’debt care pathways’ and better communication between local health and advice services [17–19]. A recent rapid assessment review of 129 eligible studies and relevant policy documents, showed that accessing financial advice services led to improved mental health, reduced stress and better quality of life [20]. Whilst there does not seem to be any specific UK research on whether mental health interventions can improve finances, some clinical trials of interventions do report on economic outcomes, although findings related to lost productivity and loss of earnings are equivocal (e.g., [21])

In qualitative research carried out following the recent (2008-9) recession, unemployed people with common mental disorders reported low levels of satisfaction with support provided by jobcentres in the UK. It was found that advisors and claimants were reluctant to discuss mental health, despite its impact on job-seeking and employment prospects [22].

In a previous qualitative, UK study we investigated the experiences of people whose self-harm was precipitated by job loss, financial difficulties and problems with welfare benefits [10]. We found that economic hardships resulting from the recession and austerity measures accumulated or acted as a final straw to trigger self-harm, often in vulnerable individuals. Our aim in this report is to draw on a wider sample, including service providers and individuals who had not self-harmed, to further understand and describe the experiences of people with financial, employment and benefits difficulties as they sought help for their problems and for the consequences of their difficulties on mental health.

## Methods

We recruited individuals for 1:1 interviews and focus groups from two cities, one in the North, the other in the South West of England. Drawing participants from these two cities enabled us to investigate the effect of economic hardship and the challenges obtaining support in settings that were likely to have different statutory and voluntary sector service support available. Following the recent recession both cities had experienced 60% rises in unemployment between 2007 and 2012 (https:/www.nomisweb.co.uk).

Interviews were carried out between September 2012 and January 2015, a period when some economic indicators were improving but when austerity measures in the form of welfare cuts continued to be introduced. These measures included restrictions to those receiving tax credits, reassessment of eligibility of out-of-work disability benefit (2010), the introduction of a maximum benefit cap (2013), the implementation of an extra room supplement for those in social housing (the ‘bedroom tax’) (2013) and introduction of Universal Credit leading to reassessment of those claiming Disability Living Allowance. Some of these changes have since been shown to adversely affect suicide rates [7].

### Recruitment of service providers

Contact was made by MB, JC and CW with thirteen health and social service providers from the voluntary and statutory sector in the two cities. The organisations included: Samaritans - a charity providing emotional support to suicidal individuals (people from branches in both cities were interviewed, including a group of outreach workers who spent a day/week in a local Job Centre), a debt advice service, a housing association, an Improving Access to Psychological Therapies (IAPT) provider, a hospital psychiatric liaison team, a union, an organisation providing support back in to work for people with mental health problems and/or learning disabilities and Job Centres in both cities (Table 1). Three organisations declined to participate.

**Table 1:** Organisations from which service provider staff were recruited and numbers of interviews conducted

Organisation	Number of staff interviews or focus groups
Samaritans (City 1)	1
Samaritans outreach team (City 1)	1 focus group (*n* = 7 participants)
Samaritans (City 2)	1
Housing association (City 1)	2
Psychological therapies provider (City 1)	1
Psychiatric Liaison Team (hospital City 1)	2
Debt advice centre (City 1)	1 focus group (*n* = 5 participants)
Union (City 1)	2
Support for people with MHP/learning disabilities (City 1)	1
Job Centre (City 1)	3
Job Centre (City 2)	2

### Interviews

One-to-one interviews were conducted by MB, CW and JC with front-line staff in all organisations in private rooms at their workplaces. Focus groups were held with staff from a debt advice centre (MB, CW) and with outreach workers from the Samaritans (MB). The topic guide covered the financial and welfare problems that users brought to the organisations’ attention (e.g., debt, benefit, job and money worries), their views about the kind of support the staff were able to provide, the service users’ reactions to their help, and their suggestions about unmet need among people struggling with economic hardship.

### Recruitment of service users

We wanted to explore the experiences of people with a range of responses to financial hardship; those who were struggling financially but who had not self-harmed as well as those who reported self-harming as, in part, a response to these difficulties. Staff aided recruitment of service users within the above organisations by giving information about the aims of the study to eligible service users and asking whether they would be interested in taking part. In addition, recruitment posters were displayed within some organisations, targeted at potential participants aged between 18 -65 as follows: (wording: “Have you been affected by the recession? Would you take part in a completely confidential interview? We would like to hear from you if: you have lost your job or, are struggling to stay in work or, you are struggling to make ends meet because of the recession or benefit changes”). Posters included contact details of the research team. Participants were offered a £20 shopping voucher as compensation for their participation.

Additional targeted sampling of participants was also carried out through a local business organisation, an advert in a local newspaper and posters displayed in work programme offices in order to recruit business owners and younger people affected by the economic downturn. In a young parents support centre, staff identified service users who would be interested in taking part in the study; they also advised that participants would be more comfortable taking part as a group. A focus group was organised on a day when most participants would be available.

Alongside information from community and service providers, data from patients who had self-harmed due to employment, financial and benefit difficulties and presented to Accident & Emergency Departments in the two cities were also used. Interviews took place between 2 weeks and 10 months after the self-harm episode. Information on the recruitment of this self-harm group has been described elsewhere [8]. The researcher completed the Suicide Intent Scale [23] for each participant following their self-harm which is used to indicate low, moderate, high and very high suicidal intent [24].

### Interviews

Interviews/focus groups were conducted by MB, JC and CW in a range of settings including the participants’ home, workplace, local café, university premises or community centre and lasted between 45 min and 2 h. All participants provided written consent for the interviews to be audio-recorded. The topic guide included questions about: their current situation and debt, benefits, job and money worries; support they had sought and why; their assessment of the value and usefulness of that support; and support or help they felt they still needed.

### Analysis

Data collection and analysis continued concurrently according to the constant comparison methods of grounded theory [25]. Interviews were audio-recorded and transcribed verbatim. Data relating to the first few interviews were analysed by detailed scrutiny of the transcripts to identify common themes, which were then coded (MB). A coding comparison exercise then took place with other members of the research team (JD and DG). Codes were refined, focused or altered and a coding framework developed. This framework was then used to code further, similar ‘sets’ within the clinical sample: older men and younger men, older women and younger women. The ‘sets’ in the community sample included: the self-employed; business owners; those facing redundancy and those seeking work and on benefits. Service provider data were analysed as a separate set. Data were examined for similarities and differences within themes and across sets. Sampling continued with the aim of achieving data saturation i.e., when new themes no longer emerged from the data.

## Results

Ten voluntary and statutory organisations in two cities agreed to help recruitment of participants. Experienced staff from each of these organisations were interviewed (*n* = 27) with 15 one-to-one interviews, a focus group of 7 (outreach workers) and a focus group of 5 (debt advice).

The ‘community’ sample (Table 2) comprised twenty-two participants - including one focus group of 5 young parents - with self-reported financial, benefit or employment problems due to the recession. The participants who had self-harmed consisted of interviews with nineteen people who had self-harmed and had financial worries. The samples included equal numbers of men and women and were broadly similar in demographics with ages ranging from 19 to 59 years. The characteristics of the sample reflected a range of circumstances in terms of partners, children, housing types and ownership, employment and social class/education. Whilst the community participants had not self-harmed, some were in considerable distress about their financial circumstances. Over half (10/19; 53%) of people in the clinical sample had self-harmed with high or very high intent and most (14/19: 74%) had previously self-harmed.

**Table 2:** Characteristics of the clinical (participants who had self-harmed) and community sample of participants and common services accessed

	Clinical (*n* = 19)	Community (*n* = 22)
Demographic characteristics		
Gender		
Male	9 (47%)	11 (50%)
Female	10 (52%)	11 (50%)
Age group		
Under 30	5 (26%)	8 (36%)
31-40	5 (26%)	2 (9%)
41-50	5 (26%)	5 (23%)
51-60	4 (22%)	7 (32%)
Employment status		
Employed	6 (32%)	8 (36%)
Unemployed: receiving Employment Support Allowance (ESA)	7 (37%)	1 (5%)
Unemployed: receiving Job-seekers Allowance (JSA)	5 (26%)	11 (50%)
Other (carer, income support)	1 (5%)	2 (9%)
Housing		
Type of housing		
Social housing	6 (32%)	9 (41%)
Private rental	5 (26%	4 (18%)
Lives with parents	4 (21%)	2 (9%)
Owns home/paying mortgage	3 (16%)	6 (27%)
Other (hostel/staying with friends)	1 (5%)	1 (5%)
Living with		
Family (1+ member of immediate family)	5 (26%)	10 (45%)
Partner	6 (32%)	4 (18%)
Alone	7 (37%)	7 (32%)
Other	1 (5%)	1 (5%)
Services accessed		
Job Centre/benefits agencies	14 (74%)	13 (59%)
Free debt advice/CAB/MIND	5 (26%)	13 (59%)
Health services (GP/Counselling)	16 (84%)	19 (86%)
Suicide intent scores		
0-6 (low intent)	0	
7-12 (moderate intent)	9	
13-20 (high intent)	8	
> 20 (very high intent)	2	
Previous self-harm		
Yes	14	
No	5	

The main themes and findings are presented in Table 3.

**Table 3:** Summary of main themes and findings

Themes and Subthemes			
Theme 1 Service Provision	Theme 2: Informal Support	Theme 3: Unmet Need Sub-themes:	Theme 3: Mental Health
Sub-themes:			
-Employment and benefit agencies		Practical Guidance through system	
-Independent/charity services		-Benefit and debt information	
-Health services		-Co-ordinated services	
Main findings within each theme			
Most participants highlighted that accessing services could be difficult	Participants who had self-harmed reported fewer sources of support, and less supportive social networks than the community sample.	All groups indicated that practical help for financial and benefit issues would have helped/would help – especially the clinical group.	Participants who had self-harmed reported a stronger belief that they should be self-reliant in the face of economic and mental health difficulties than the community sample.
Free debt advice, when it could be accessed, was considered the most useful service	Participants who had self-harmed reported more difficult circumstances such as benefits changes or sanctions.	All groups wanted straightforward and clear information about services available and how to access them. Co-ordination between services would help.	
The community sample reported more knowledge of how to access debt advice (as expected) than the participants who had self-harmed – although both groups had sought similar types of help		All groups felt that help for current and past mental, emotional and physical difficulties was necessary	

These themes will be presented in more detail below. The names of participants have been changed to protect anonymity.

### Service provision

The most common (non-health services) accessed were to do with employment - specifically job centres and benefits agencies - followed by other money advice-related services provided by independent or charity organisations. However, service provision was not straightforward. For example, help for appealing against benefit sanctions was only available for those living in social housing provided by the local council; information about non-statutory (non-state run) services that could help with financial difficulties was often not up-to-date; and accessing service provision was sometimes problematic due to waiting lists and funding difficulties amongst service providers, and some services had overlapping provision.

Non-statutory services struggled to provide continuous support for clients due to dependence of the organisation on short-term funding grants. Referral routes to or between services were not clear, and knowledge and information about available services varied. Service providers were particularly concerned about their ability to care for people appropriately with such confusion inherent in the system and multiple changes being made. For example:

‘There’s an awful lot of worried people out there at the moment because the benefit systems changing on an almost daily basis but we don’t have the expertise or time to find out what’s what’ (Simon, Employee of Social Enterprise helping vulnerable people back into employment)

Service providers were also concerned by the gaps and overlaps in service provision:

Carrie: It could be quite confusing because you’re not sure what another organisation is already doing for someone

Joy: Confusing for the clients as well, isn’t it? (Debt Advice Staff Focus Group)

- Employment and Benefit Services (Job Centres)

Most of the clinical and community sample had contact with a Job Centre, whether they were actively looking for work and receiving jobseeker’s allowance (JSA; a benefit for those judged healthy enough to work), employment support allowance (ESA; a benefit for those whose capacity to work is judged to be limited by their health) or were trying to earn a living through self-employment. They reported overwhelmingly negative experiences of looking for work and signing on with the Jobcentre. Staff were often considered rude and unhelpful, which added to the service users’ feelings of worthlessness due to their unemployed status and the implied stigma.

‘I found they were rude and they’d look down their nose at you… You already feel badly enough about signing on, and then these people make you feel even worse. Don’t like it.’ (Angie, 40s, JSA, Community)

‘It’s just like every day going down the job centre and holding the telephone [trying to get emergency loans] and ‘your call is in the queue’ for an hour every day and going ‘where’s my giro, where’s my money?’, and then you don’t get it and I go ‘well what am I meant to do then?’ … So that was like really stressful’. (Jeff, 50s, JSA, Community)

‘I was applying for jobs, my CV was up to date, but they kind of make you feel, like the adviser I had made me feel like I wasn’t trying hard enough. They were trying to send me to interviews in (areas away from city) and I didn’t’ have any money and I’m like ‘well how am I going to get there?’ (Zoe, 40s, Participant who self-harmed)

‘One issue that I do have finding a job it’s almost like they’re so quick to just push you into one that they’re not really considering if you’re in a job that’s going to be really beneficial to you but they’re trying to meet a quota to say ‘ok, we have this many people down, we’ve put back into work’, but by the end they might have that job for a month and then they’re back in that situation (without work) so is it quality or quantity we’re looking at here?’ (Mia, 30s, JSA, Community)

A member of the Job Centre staff acknowledged that the system could be inflexible but also placed staff behaviour within the context of a pressurised system where quotas and work capacity made it difficult for staff:

‘I think in this sort of work you get people – not that they aren’t sympathetic – but they’re ‘it’s black and white, ‘he hasn’t looked for a job, we’re stopping the money’ but then you’ll get other people that will look a little bit further and say ‘well is there an underlying problem?’ And try and signpost people but again for signing on they’ve [staff] got four minutes per person to check their job search, make sure they’re doing everything they’re doing, put notes on the system’ (Penny, Job Centre)

There were examples given by service providers of the impact of welfare changes on vulnerable people. Whilst most agreed that change was needed, it was felt that the way it was being implemented was potentially dangerous for service users with mental health problems. This was perceived to be the case particularly for vulnerable service users moved from ESA to JSA through the Fitness to Work test and consequential drop in income, even though they did not feel mentally or physically able to manage the onerous process of job-seeking to receive JSA benefit:

‘I’ve one particular chap who was attending an overcoming depression group here, who was whispering to me ‘I haven’t worked in many years but I’d quite like to work. Can we look at a CV?’ He was very, very quiet, very tentative. We started to do that work, it was very positive, and then he had this letter saying’ we’re going to re-assess your benefits’ and he just- he flipped and he- it really exacerbated his symptoms. He was- he was talking suicidally when he had not done so in many, many years…I understand its necessary and I think there’s a lot of positives to it but how it’s being implemented is very - it’s really affecting this particular group badly’ (Neil, IAPT Provider)

As has been described elsewhere [8], participants’ talked about how losing work and being unable to keep up with debt or rental payments had led to them losing their homes. This was a view repeated and reinforced by the number of cases seen by service providers:

‘We’re getting people all the time, even men coming crying and saying ‘look we’re going to lose our house, what can we do?’ and there’s nothing, you know’ (Penny, Job Centre)

b. Independent/charitable services

The main type of support accessed by participants for their debt and benefits difficulties were free advice centres such as Citizen’s Advice Bureau. These services were talked about more by the community participants who generally had more knowledge of free debt advice organisations and how to access them than participants who had self-harmed. Clinical participants were most likely to have accessed services only after their self-harm.

Participants from both groups described a similar range of debt and/or benefit problems. Many were in debt to utility companies or with council tax. The narratives of participants who had self-harmed more often included experiences of benefits sanctions (benefit payments stopped due to infractions), or being moved from ESA to JSA.

The debts of most community/clinical participants were not particularly large e.g., most often < £1000 ($1200/Euros), but as they described being on very restricted incomes – often having to choose between spending money on food or heating – any amount of debt very quickly became unmanageable. The combination of very low income and debt was extremely stressful. Threatening letters from organisations they owed money to made people fearful and confused and could prevent them actually accessing support they were entitled to. Participants wanted help understanding and managing a situation that had become frightening when they felt they had nowhere else to turn.

Participants found out about debt advice centres through a number of routes. If they were in social housing (subsidised housing provided by local councils or charities), they had contact with housing or tenancy support officers. Often people had heard of the centres by word-of-mouth. The support received through free advice centres – particularly debt advice - was considered useful by the majority of participants. Support that was particularly valued was when staff addressed or translated dense and confusing communications from the benefits agencies, banks and creditors; acted as advocates between the participant and organisations with whom they were in debt; or gave information about how to prioritise and manage debt:

‘I think going to [the debt advice centre] and them contacting the people that I’m in debt with, they helped definitely because you get the letters and sometimes I don’t know what they’re talking about. The letter from DWP I got like three or four different letters between the space of two days, it had different amounts that they were taking out, um, it was just all confusing…it’s a 0845 number so that’s just running up my mobile bill’ (Tash, 30s, ESA, Community)

‘Before [going to debt advice] the bills were just coming through the door and ‘cos I was so stressed I was just like sticking them on the side and not even opening them and sat in my flat thinking ‘oh my god, the bailiffs are going to come round’ etcetera and then I thought ‘oh I’ve got to do something about this’ but I couldn’t face it myself so I just took it to someone else (laughs) to do it for me basically…it took like a couple of months to get it all sorted.’ (Maddie, 30s, ESA, Participant who self-harmed)

Some self-harm patients were not aware of these services until after they had reached crisis point and self-harmed:

‘[the] nurse referred me to MIND. They - they really were, they did so much. They [helped me to fill in the forms to appeal the benefit decision] did so much. One of those things that unless- unfortunately if I didn’t try and commit suicide I wouldn’t have known about them’ (Joe, 30s, ESA, Participant who self-harmed)

However, the waiting times for services could sometimes be lengthy and the advice given not always helpful:

‘I had so many people say that they (debt advice centre) helped them and then they can’t help me, I don’t know if it’s because it’s a business or what. Basically she told me to hide ‘because none of your creditors know where you are so - I haven’t told them and I won’t tell them’. How long can you hide for?’ (Angie, 40s, JSA, Community Sample)

c) Health services

While most participants had contact with their General Practitioner (GP), participants who self-harmed tended to describe accessing help and support from their GP about their financial worries only after their self-harm.

Participants commonly spoke about being debilitated with depression, stress and anxiety with concurrent sleep problems because of their debt, benefits or financial difficulties. Medication was the support usually provided to participants from their GP. Whilst antidepressants or sleep aids were sometimes considered a necessity to help them function, most participants seemed to want the offer of a talking therapy to help them manage their situation or at least to be listened to. People had mixed views of their interactions with their GP.

‘I think since this I had - my doctor’s been giving me like antidepressants and temazepam to sleep at night and stuff like that and I think I’m more- really depressed.’ (Angie, 40s, JSA, Community Sample)

‘…they said exercise is really good for depression and they give me free tickets to go down the gym and that’s alright. Apart from that, no [support]…they said I wasn’t that bad to actually get referred to the inner city mental health team or something like that - basically you got to be pretty suicidal really then they’ll put you in that programme.’ (Jeff, 50s, JSA, Community Sample)

‘I did make an appointment to see her, for a sick note, but I did want to speak to her as well but she was like ‘you don’t need to come back, I’ve already printed off the sick note and it’s at reception’ (Tash, 30s, ESA, Community Sample)

Positive experiences of GPs tended to be because of a consistent, supportive relationship between patient and practitioner, or participants feeling that they had time to talk, were properly listened to, and given useful information about other psychological support services.

‘I go to see my GP at the moment. Yeah, he’s (pause) he’s an alright bloke to be honest. He gives me ways of dealing with things…he’s giving me numbers for Off the Record and LIFT which are counselling classes. Um, (pause) he put me on antidepressants… he sees me on a regular basis so it’s quite good for keeping in touch.’ (Ash, teens, ESA, Participant who self-harmed)

‘Also the doctor up there I see, Dr J, up at (surgery) and he’s absolutely brilliant. Absolutely brilliant. Oh I go - I’m supposed to go and see somebody, is it LIFT or something?....’ (Tommy, 40s, Participant who self-harmed)

### Informal support

There were mitigating effects – both practical and emotional – of having a family or friendship support network. Participants who had people they could rely on for material assistance talked more about being able to cope with the stresses of their financial situation. This assistance included meals, help paying bills or helping them to socialise when they would otherwise be unable. Emotional support given by social networks, which often but not always, came with practical help, also enabled participants to manage their lives. These types of informal supportive networks were described mostly by the community participants:

‘I’m hunting [for work] or sleeping people’s places, like two days there or I’m back, three days there. I spend time on friend’s places, you know, because electric and all those things.’ (Manbwe, 40s, self-employed, Community)

‘I’ve got music, yeah… punk rock, it is a lot of people like and most of us are not rich, but you are… it’s like people stick together and support each other so that’s good. .. ‘Yeah, it’s being part of a union in a way, you know. That helps to get through a crisis, like if you have some sort of union.’ (Jeff, 50s, JSA, Community)

‘I mean I wouldn’t have been able to cope recently if I didn’t have my mum around and she hasn’t got much money, I am in debt to her at the moment which I hope I can earn enough in the summer to pay her back.’ (Jo 30s Self-employed Community)

‘That’s what I said, we have a good- we have a really good friend network and being women, I think women talk a lot-’(Sheila, 60s, Facing Redundancy, Community)

‘It’s like an extended family down here [community centre]. Everyone gets on so well, people have their differences but everyone’s – it is just like an extended family. It’s good’ (Nic, 20s, JSA, Young Parent Group Community)

In stark comparison, there were few descriptions of supportive networks amongst those who had self-harmed; these participants largely described intense feelings of isolation, often in the context of difficult family relations (historic and current), or estrangements which had often been exacerbated by their financial worries. For those that had family and friends close by, they were not necessarily a positive or supportive influence.

‘We currently don’t have any [family or friends] - well, I’ve got one friend but he’s not reliable ‘cause he’s, um, he’s an alcoholic.. My family are non-existent, um. I’m one of seven children but, um, they all went into foster care but I stayed with my mum and I grew up as an only child but no close family now.’ (John, 30s, ESA, Participant who had self-harmed)

‘We’re not a family where people really talk about stuff. It’s one of the reasons I think I have a problem because my mum and dad, if I started crying in front of them they both wouldn’t be able to- they’re very like- they don’t do that kind of thing’ (Zoe, 40s, Participant who had self-harmed)

‘It was just a load [of stressors]. Yeah [I felt] just completely and utterly alone, nowhere to turn’ (Joe, 30s, ESA, Participant who had self-harmed)

Service providers confirmed the importance of social networks in providing a safety net for vulnerable clients:

‘They’re always the most heartbreaking cases [the ones with no-one]. They’re always the most distressed, you know, I always leave feeling most upset about those because when anyone’s got some family support you kind of know they’re going to be alright somehow. You know, they’ll be distressed but they’ve got that support network, but without that it’s awful.’ (Tess Debt Advice)

### Unmet need

The service providers, and the clinical and community participants all expressed similar requirements in terms of unmet needs, albeit in slightly different ways according to their circumstances. Service providers’ views were based on their long-term experiences with servicer users; the clinical groups’ opinions were influenced by experiences before and after their self-harm, and the community participants had sometimes had their needs met, although were not necessarily satisfied with the services they had received.

The most common need expressed was for practical help with benefit and debt problems. It was acknowledged that mental health support was a requirement for those with complex present and past emotional difficulties. Clear access and referral to both these types of services was also required. However, these needs have to be addressed within the context of the extreme self-reliance expressed by many of the clinical participants in comparison to the community group.

All participants felt they would have benefitted from extra help when they were at their most stretched due to financial troubles. Furthermore, some service providers were deeply concerned about the needs of service users that were not being met, hampered by the complex benefits system, and highlighted the impossibility of making ends meet:

‘I had a lady yesterday and she didn’t go to the work programme so they’d stopped her money for a month …well she was just absolutely sobbing when she came in and she said’ It’s got too much, I just can’t cope’, so she went to the doctor and she came back with a sick note and I did give her a referral to get a food parcel because she’s got a child that’s seven’ (Penny, Job Centre)

‘We do I think get more calls from people who either they’re worried because they’re being denied benefits or they’re having to go along for interviews and go through this process of being reassessed and they’re terrified that their money is either going to be cut or withdrawn completely or other people where that has happened and they are really struggling’ (Julia, Samaritans)

‘A really big thing as well is going to be the, um, bedroom tax so people living with more bedrooms than they need are going to have to top up at least fourteen pounds a week out of their benefit if they want to stay in their property, so if somebody’s just on seventy one pounds anyway that’s a huge percentage to find.’ (Joy, Debt Advice)

- Practical support needed: guidance through the system

The most important support that people said they needed was practical advice to help them move on from their immediate crisis and manage their financial situation. For the most part, this support had not been found by participants who had self-harmed, nor addressed by the agencies they had come into contact with.

When people were at their most vulnerable they found it most difficult to access the support they need. Participants indicated that counselling or listening services were not the only answer to their problems; it was concrete financial and debt guidance that would particularly help. Implicit in many of the accounts was the desire for support that would help with practical aspects of their situation. This wish for pragmatic solutions was most deeply expressed by those who had self-harmed, but also by those in crisis amongst the community sample:

‘Someone that knows all the benefits you’re entitled to, ‘cause I didn’t realise I was entitled to more ESA. I thought I was getting the set rate. Advice as well, yeah, ‘cause that’s what you need. You need advice not someone to just go ‘yeah, yeah, there there, it’s alright’. It’s not what you need, is it?’ (John, 30s, ESA, Participant who had self-harmed)

‘I think if you’ve got mental health problems and you’re having problems regarding [benefits] your GP or something should go ‘listen you’re going through a really bad time but there is an organisation out there that deals with this kind of thing specifically and they’re really good’ because if I [hadn’t] got the referral from the nurse, I’d be just as lost now as I was’ (Joe, 30s, ESA, Participant who had self-harmed)

‘I need to sort out my finance, I need to sort out this problem with the money that I owe, that’s what is making me like this [very depressed]. I could talk to somebody forever every day for a whole month but I’m still going to have this problem…I really should be going out there and sorting this out but I just can’t face it. Just can’t do it. (tearful)’ (Angie, 40s, JSA, Community)

For participants with literacy problems, or where English was not their first language, practical guidance through the system and help with form filling, particularly in a face-to-face manner was indicated to be the most useful. The absence of this simple support added to the sense, despite the high targets set for job-seeking, there was little concern for the individual:

‘Help, yeah, with writing and fill form- well usually they sit there- it’s not their job to help people to fill form - usually that what they say. Probably is too many ticking boxes so they can’t be bothered sitting down to help.’ (Manbwe, 40s, self-employed, Community)

‘I’m totally rubbish at filling out forms so that’s why I asked them for help and it just didn’t arrive. It always takes me ages to do them. I never know how to word things, I never know what to write. [I‘d like someone to] guide me through it.’ (Matt, 40s, ESA, Participant who had self-harmed)

b) Benefits and debt information

Service providers pointed out the gaps in the statutory support networks for people who get into financial difficulties:

‘If people are in [named] funded accommodation the organisation gives money for people for the housing association to provide support – these people are the lucky ones, if they’re having problems we can refer them to the city council welfare rights and they provide casework and do appeal for you…but there are a whole group of people who are not in [funded accommodation] who just have to go to CAB’ (Jackie, Tenancy Support Officer)

The Samaritans Outreach team focus group felt that debt advice was essential – yet often not offered or accessed - for the population they saw:

‘For the financial, economical issues that we’re talking about because actually they feel left alone, bereft of ideas – they haven’t been trained to look after money often’ (Tom, Samaritans volunteer doing outreach work in a Job Centre)

There was a clear sense from all those in contact with the benefits system of what was needed and missing: timely and clear information. Participants described the difficulties involved in trying to access any information and feeling as if they had nowhere to go where they could find out what they were entitled to and how to get it; nevertheless, they could be sanctioned (have their benefits reduced or stopped) for not giving the correct information themselves. This was particularly true for those participants who had set up in self-employment or tried to start to start a business:

‘The other main issue I have with any of the benefits agencies is that they don’t give you enough information and then when you don’t fit in they beat you with it…if you don’t know what to look for how are you supposed to ask? So the information is very low, being able to get in contact with them is very difficult.’ (Jo, 30s, self-employed Community)

‘I don’t know what benefits I’ve got coming in and when they’re supposed to be coming in or what should I be claiming? What am I allowed? There’s a big failing there. There’s not the support, you have to go out and find it yourself which takes time and like I say, you’re just a number shall we say’ (Mark, 50s, self-employed, Community)

‘When I had my first baby I was- I didn’t have a clue what I was entitled to… my nan was going through the money she said ‘why was I not claiming for tax credits’?. I said ‘no, what’s that?’, I think it was £56 a week, it was a huge amount, especially with having a baby and only having just over a hundred pound coming in a week.’ (Emma, 20s, JSA, Community)

c) A Co-ordinated approach

To address the piecemeal, confusing and delayed services they had experienced, service users and providers suggested a form of co-ordinated approach, or central number that could be contacted by staff who could then refer or signpost an individual to the appropriate service, avoiding waiting times and distress as the financial situation and fears worsened:

‘Well, a co-ordinated approach, whether that be from the city council gets together, gets them all together and you have one line number or something and it gets directed to the right party. ‘Cause action needs to be done almost immediately, not wait. The longer you wait, day by day things get worse. Emotionally, stress wise, etcetera, and the finances as well.’ (Mark 50s self-employed Community)

‘It would be good that if you were laying someone off, that there was a starting point if you like – for everybody, that was well known. This is where you go, maybe that’s attached to the job centre, maybe that could be attached to the general practitioner. Something that was clear, you know, 118 118… a one-stop’ (Jack, a Union representative)

### Mental health – self reliance

Rather than help-seeking, those who had self-harmed were more likely to talk about how only they could have helped themselves or how difficult it was to talk about problems either because of cultural expectations of masculinity, not wanting to burden others, or because they were too busy simply trying to survive.

It was of note that this kind of self-reliant talk held true for men and women:

‘the thing is I find it hard to talk … ‘cause I’m ex-forces and I always think, you know, you just deal with this stuff yourself. Get it sorted yourself, that was what’s ingrained into you while you’re in the forces and told to ‘man up’ I find it very hard to seek professional help..’ (James, 40s, Working, Participant who had self-harmed)

‘The kids had gone back to their mum the day before and, it was just a load. Yeah, just completely and utterly alone, nowhere to turn. I mean it’s all good living with my mum and that but she’s got enough problems to sink a ship so I try not to push all my problems out onto other people. I try and deal with them best I can myself. I could have stopped myself to be honest by actually opening up to people instead of bottling it into myself.’ (Joe, 30s, ESA, Participant who had self-harmed)

‘I mean when shit things happen to you, you know you’re going to be down, so you don’t necessarily go running to the doctor or somewhere for help do you straight away, maybe…but I’m so busy trying to survive and get a job and (pause) and deal with the dole.’ (Helen, 50s, Working, Participant who had self-harmed)

‘I don’t think there’s anyone who can help me in this position that I’m in. The only solution is to get work and earn money. No-one’s going to put money in my bank account are they (laughs) and I want to work as well. So that was- I don’t think there is any help out there really for someone in this kind of situation.’ (Zoe, 40s Participant who had self-harmed)

Nevertheless, some of the participants who had self-harmed could appreciate that some sort of help would have been useful to deal with the deep-seated, historic problems they had experienced, such as domestic and/or childhood abuse or to address associated long-standing feelings of worthlessness:

‘I do think I need help because I feel I would be a much better person if I did and maybe if I did get some help I would be much more approachable by employers because maybe they see something inside me that they don’t like.’ (Debbie, 30s, JSA, Participant who had self-harmed)

Service providers felt that there was a lack of resources available for people needing mental health support, and that clients had to wait for too long:

There’s a lack of resources..just knowing that if they are referred on that they’re going to get seen quickly. We’ve got huge waiting lists. Knowing that when you withdraw support from somebody, it’s not going to be six months before they get picked up by a counselling service (Helen, Housing Association)

Waiting lists and errors could mean long delays in essential treatment. Some participants described trying to access secondary mental health care but finding their pathway ‘blocked’, either by the system or by the seeming lack of understanding by GPs of the nature and seriousness of the participants’ situation and mental health condition:

‘I started having recurring issues with my depression around January time, I started to be all worse again and I self-harmed for the first time since I was about sixteen and because I was aware that it was an issue I went to the doctors and again asked to be referred to psychology. I had an assessment a couple of months later at the GPs and they told me they’d be referring me to Cognitive Behavioural Therapy. Again never got referred’ (Lisa, 20s, JSA, Participant who had self-harmed)

‘I’ve been waiting for [therapy] - since that day [10 months]. They put me on this list but it was (area) and I said ‘that’s too far’ and they said ‘oh it’s a mistake, you’re supposed to be on the one in town’, and then a month or so ago they rung me again with an appointment and it was the (area) one again ..so no I haven’t’ (Tracey, 40s, Participant who had self-harmed)

## Discussion

Participants who had self-harmed and community participants reported common issues when accessing or attempting to access services associated with benefit, debt or employment issues. Most had sought support for financial and emotional problems. However, the provision of services was confusing and navigation through the support and benefits system difficult for many and particularly challenging if people were already vulnerable; a view reinforced by service providers working on the frontline with service users. There was a clearly expressed, shared desire amongst the three groups – participants who had self-harmed, community and service providers - for practical support to help people out of their financial difficulties. Whilst the need for help with current and past mental and emotional issues was also expressed, this support was felt to be ineffectual if the underlying cause (often financial) of their distress was not being addressed.

Our study supports work that suggests an integrated approach to relevant advice for service users through integrated working across sectors and by making existing pathways more visible and accessible [15–17]. For example, mental health services can (and sometimes do) help service users with financial problems such as challenging benefit decisions or in debt advice. However, there is not a consistent approach to integrated working across the UK.

As found elsewhere [22, 26], problems with debt seldom manifest in isolation but commonly present alongside increased vulnerability to multiple life problems. Participants in the current study often had complex difficulties and, as reported by others, action to resolve individuals’ problems had not been well co-ordinated; addressing only ‘health’ or ‘debt’ problems separately may be ineffective [16]. However, especially important to the participants in the current study was guidance in addressing their financial difficulties. ‘Person-centered’ debt advice in Ireland was found to have considerable impact in alleviating the pressure felt by service users and had long-term benefits such as reported improvements in psychological health and family relationships [27].

The findings demonstrated a need for staff in frontline Jobcentres to be better trained to recognise mental health problems. Some Jobcentres (e.g., in City 1) had staff trained in the nationally recognized Mental Health First Aid programme to address this issue. However, there is not a consistent approach to identifying mental health needs supportively across Jobcentres in the UK. Clinical staff could also benefit from training to help them identify financial and employment difficulties and refer of patients for suitable support.

The main differences between groups were to do with levels of knowledge of debt advice services and how to access them. The participants who had self-harmed had generally only found out about free debt and benefit advice services *after* their self-harm episode. These participants also differed from the community participants in their reported self-reliance in response to their money worries and difficult circumstances. The other main difference was the stark contrast between informal support networks described by both groups. In comparison to the community group, those who had self-harmed rarely reported positive relationships with family or friends and often described having no-one they could talk to or rely on.

It could be interpreted that these are not mutually exclusive findings; not having good support networks could be seen to lead by necessity to self-reliance. If you have no-one to talk to and there is no-one to advise or guide you, it is unlikely that you will seek help from support organisations. This finding is in keeping with research that highlights the overlooked contribution made by family and friends in problem-noticing and signposting to advice services, and how vulnerable people in particular rely on the physical presence of friendly others in the advice setting [28].

Community groups described higher levels of knowledge and access to debt and benefits advice, possibly as an artefact of our recruiting participants through service providers. However, community accounts also highlighted the support they received from being linked in to local networks and often described hearing about services from a friend or family, or accessing services *with* someone.

Most of the participants were not working. The benefits of employment for mental health are well established and reflect a combination of income and access to resources, as well as the psychological advantages of social roles and access to social networks and supports [29]. Connections between mental health and economic participation have often been at the forefront of government policies targeting social inclusion for a healthy, economically productive society [30, 31]. In contrast, excluded individuals experience multiple disadvantages including inadequate financial resources, limited social support and networks and poor health [30]. Our results suggest that in times of austerity the state of exclusion is easier to slip into or remain trapped in, and that inclusion is not at the forefront of government policies.

The question is how to reach people with little or no social network and who are self-reliant but struggling with finances? As has been reported from several initiatives targeting particular vulnerable groups, to give advice is challenging [32, 33]. However, it can have very positive reported effects by service users although outcomes are not always immediate or easy to measure in the standard way [11, 13, 14].

Catching people early in the trajectory of their financial struggles – be they benefits, debt, employment or a combination of difficulties – is crucial to preventing the spiral of emotions and events that can lead to extreme distress and, sometimes, self-harm. Any intervention related to finances would also need to be sensitive to mental health concerns as all participants in all groups in the study indicated that they were usually interlinked. The complex and challenging nature of the bureaucracy involved in the benefits system can actively heighten people’s sense of despair and isolation. Interventions to identify those in need and aid service users in accessing practical, accurate and free advice from the support sector could help mitigate the effects of benefit, debt and employment difficulties for vulnerable sections of society. Alongside this, the complex nature of many service users’ other difficulties would need to be acknowledged. Help in accessing mental health support is also likely to be essential in ongoing recovery and enablement.

### Strengths and limitations

The current research benefits from including in-depth accounts from service provider and user perspectives in two cities and includes the voices of those who were affected to the extent that they made high-risk suicide attempts. The findings extend understandings beyond the largely quantitative work in this area. A positive point was that data collection spanned 2012 to 2015 and not a single point in time; a period that included a number of changes to government policy.

Many participants in the community sample were accessed via the advice centres, so inevitably knew of their existence. The research would have benefitted from including people who were struggling with financial worries and not accessing help but who had not self-harmed.

### Implications

The findings from the current research suggest a number of policy implications that could be operationalised at local and national levels (Table 4).

**Table 4:** Implications for policy

Locally
• Local authorities should regularly update information on the sources of advice available for those experiencing economic hardship in the area, providing all sectors with regularly revised updates of paper or web-based summaries of these
• Practical help could be made available to help people decipher official information and guiding them to voluntary/statutory sector agencies
• Employers making redundancies could pass on information on local support agencies to at-risk employees.
Nationally
• Written communications from DWP/Job Centres could involve service users proof reading the text to make it more understandable
• Strategies to address the mental health impacts of recession and debt need to appreciate men’s (and some women’s) unwillingness to talk about problems and seek help
• Increase resources to statutory and non-statutory organisation providing help for people affected by economic downturns including training front line staff in recognising and responding to emotional distress due to financial concerns.
• Mental Health First Aid training could be given to all front-line staff
• Clinicians may also benefit from training in recognising financial and employment issues and referring on to the best support.

There is a need for Local Authorities to regularly review and update information/advice available in the area and ensure all sectors receive regularly revised updates of paper or web-based information. There is also a need for practical help deciphering official information and guiding people to voluntary and statutory sector agencies. Employers making redundancies should have access to information on local support agencies to pass on to their workers.

Nationally, all written communications from the Department of Work and Pensions (DWP)/Jobcentres could involve service users in proof reading the text. It should also be remembered that the readers of DWP communications are disproportionately drawn from people with lower levels of education and sometimes limited reading and writing skills.

Strategies to address the mental health impacts of recession and debt need to include initiatives to address men’s (and some women’s) general unwillingness to speak about their emotional health problems and to seek help.

There is a need to increase resources to statutory and non-statutory organisations providing help for people affected by economic downturns in a timely way. Structural changes might include training of front line staff in recognising and responding to people who are emotionally distressed due to their financial situation, or in need of debt advice. Jobcentre staff could also be given more time in which to advise people seeking and applying for jobs and accessing social security benefits and to provide mental health first aid. As findings suggest that people attending clinical services also may not be receiving the help they need, the ongoing needs of these professionals could be addressed through provision about the link between mental health and financial worries, and in building confidence and knowledge about referral to relevant local support services, such as free debt advice organisations.

## Conclusion

Economic hardships resulting from the recession and austerity measures can accumulate or act as a trigger to self-harm, often in vulnerable people. Participants who had self-harmed and community participants reported common issues when accessing or attempting to access support services associated with benefit, debt or employment problems. The provision of services was confusing and navigation through the support and benefits system was difficult. There was a clearly expressed desire amongst participants for practical support to help people out of their financial difficulties. Findings support an integrated approach to relevant advice through integrated working across sectors, along with frontline staff training to recognize and address mental health problems. Early interventions to identify those in need and aid service users in accessing practical, accurate and free advice from the support sector could help mitigate the effects of benefit, debt and employment difficulties for vulnerable sections of society. Further research is needed to evaluate exisiting interventions for vulnerable people and to develop, pilot and evaluate interventions tailored to their needs. Together, these steps could help alleviate the burden of debt and mental distress felt by the most vulnerable members of society, especially at times of economic recession.

## Abbreviations

DWP: Department of Work and Pensions (Government Department responsible for welfare)
ESA: Employment Support Allowance (welfare benefit)
GP: General Practitioner (Medicine)
JSA: Jobseekers Allowance (welfare benefit)
